# IL-10 Expression-Inducing Gut Bacteria Alleviate High-Fat Diet-Induced Obesity and Hyperlipidemia in Mice

**DOI:** 10.4014/jmb.1912.12014

**Published:** 2020-01-09

**Authors:** Hye-In Kim, Soo-Won Yun, Myung Joo Han, Se-Eun Jang, Dong-Hyun Kim

**Affiliations:** 1 Department of Food and Nutrition, Kyung Hee University, Seoul 02447, Republic of Korea; 2 Department of Life and Nanopharmaceutical Sciences and Department of Pharmacy, Kyung Hee University, Seoul 02447, Republic of Korea; 3 Department of Food and Nutrition, Eulji University, Seongnam 13135, Republic of Korea

**Keywords:** Gut bacteria, IL-10, obesity, liver steatosis, colitis.

## Abstract

In the present study, we examined the effects of interleukin (IL)-10 expression-inducing bacteria *Bifidobacterium adolescentis* HP1*, Lactobacillus mucosae* HP2*,* and *Weissella cibaria* HP3 on high-fat diet (HFD)-induced obesity and liver steatosis in mice. Oral gavage of HP1, HP2, and HP3 reduced HFD-induced bodyweight gain, triglycerides, and total cholesterol levels in the blood and liver. They also suppressed HFD-induced colitis and the fecal δ ,γ-Proteobacteria population. Of the tested bacteria, HP2, which most potently inhibited IL-10 expression, also suppressed HFD-induced bodyweight gain, liver steatosis, and colitis most effectively. These findings suggest that IL-10 expression-inducing gut bacteria can suppress obesity and liver steatosis.

The excessive consumption of a high-fat diet (HFD), a main environmental factor for obesity, causes abnormal fat accumulation in adipose tissue and the liver, which secretes inflammatory adipokines such as tumor necrosis factor (TNF)-α [1-3]. Therefore, obesity is closely associated with inflammation. Long-term HFD feeding causes gut dysbiosis: it increases the Proteobacteria population in gut microbiota [4, 5]. HFD feeding also induces TNF-α expression while suppressing IL-10 expression in innate and adaptive immune cells via the regulation of NF-κB activation, leading to gastrointestinal inflammation [5, 6]. However, IL-10 suppresses NF-κB activation in the adipocyte [3].

Lactic acid bacteria (LAB), including lactobacilli and bifidobacteria, have been reported to support the maintenance of gut microbiota balance in humans and animals, regulate host immune response, and have hepatoprotective, anti-colitis, and anti-obesity activities [7-9]. *Leuconostoc mesenteroides* subsp. *mesenteroides* SD23 mitigates body weight, liver steatosis, and liver IL-10 expression in HFD-induced obese rats [10]. *Lactobacillus sakei* OK67 alleviates HFD-induced blood glucose intolerance, obesity, and IL-10 expression in mice [6, 11]. Nevertheless, studies on the interplay between obesity and IL-10 expression have not been conducted thoroughly.

Therefore, we selected IL-10 expression-inducing *Bifidobacterium adolescentis* HP1*, Lactobacillus mucosae* HP2*,* and *Weissella cibaria* HP3 from a human fecal bacterial strain collection and examined their effects on HFD-induced obesity and liver steatosis in mice.

Gut bacteria were cultured in De Man, Rogosa and Sharpe (MRS) broth (1 L, 37°C, 24 h), centrifuged (5,000 *g,* 25 min, 4°), and washed with saline, as previously reported [6]. Collected cells were suspended in phosphate-buffered saline (PBS, for in vitro study) and 1% dextrose (for in vivo study).

Mice (male, C57BL/6, 19-21 g, 6 weeks old) were purchased from Orient Bio Inc. (Korea). They were kept under controlled condition (temperature, 20-22°C; humidity, 50 ± 10%; and light/dark cycle, 12 h) and fed with standard laboratory chow and water ad libitum. The mice were also acclimated for 1 week before the experiment. Animal experiments were ethically performed according to the NIH and University Guideline for Laboratory Animals' Care and Usage. Animal experiments were approved by the University Committee for the Care and Use of Laboratory Animals (IACUC No. KHPASP[SE]-18-003).

Macrophages were isolated from the peritoneal cavity of mice according to the method of Jang *et al*. [12]. To select IL-10 expression-inducing gut bacteria, macrophages (1 × 10^6^ cells/well) were treated with gut bacteria (1 × 10^5^ colony-forming unit [CFU]/ml) in the absence or presence of LPS (100 ng/ml) for 20 h. IL-10 expression levels were assayed using an ELISA kit.

To evaluate the anti-obesity effects of LAB, mice were randomly divided into six groups, LFD, HFD-V, HFD-HP1, HFD-HP2, HFD-HP3, and HFD-GA. Each group consisted of 10 mice. The preparation of obese mice and investigation of the anti-obesity effects of gut bacteria were performed according to the method of Jang *et al*. [6]. Mice in the LFD group were fed an LFD for 4 weeks and thereafter the vehicle was orally administered (gavaged) with LFD for 4 weeks. Mice in the HFD-V group were fed an HFD diet for 4 weeks and thereafter the vehicle was orally administered with HFD for 4 weeks. Low-fat (D12450B) and high-fat diets (D12492) (Supplement Table S1) were purchased from Research Diets Inc. (USA). Mice in the HFD-HP1, HFD-HP2, HFD-HP3 and GA groups were fed an HFD diet for 4 weeks and thereafter simultaneously gavaged HP1, HP2, HP3 (1 × 10^9^ CFU/mouse/day), or Garcinia [13] (GA, 160 mg/mouse/day) with HFD for 4 more weeks, respectively. Mice were sacrificed 20 h after the final treatment with test agents. Blood, livers, and colons were removed and stored at −80°C until used for ELISA and immunoblotting.

Aspartate transaminase (AST), alanine transaminase (ALT), triglyceride (TG), total cholesterol (TC), and high-density lipoprotein cholesterol (HDL) levels were assayed in the blood and liver according to the method of Jang *et al*. [6,14]. The LPS content in the blood and feces was assayed by using an LAL assay kit according to the method of Kim *et al*. [5]. Myeloperoxidase activity and immunoblotting analyses were performed according to the method of Jang *et al*. [14]. Quantitative real-time polymerase chain reaction (qPCR) was performed according to the method of Lee *et al*. [15]. The detailed methods are described in the Supplement. Experimental data values are indicated as mean ± standard deviation (SD). Statistical significance was determined using one-way ANOVA followed by Duncan’s multiple range test (*p* < 0.05).

We examined the effects of human fecal bacteria on the IL-10 expression in macrophages (Fig. 1). Of these bacteria, HP1, HP2, and HP3 significantly increased IL-10 expression in macrophages stimulated with or without LPS. And of these, HP2 most potently induced IL-10 expression.

HP1, HP2, or HP3 was gavaged at a dose of 1 × 10^9^ CFU/mouse/day in HFD-induced obese mice and their anti-obesity effects were examined (Fig. 2). HFD-feeding significantly increased the bodyweights in mice compared to feeding with LFD. Oral gavage of HP1, HP2, or HP3 significantly reduced HFD-induced bodyweight gain and epididymal fat pad weight. HFD feeding also induced TC, TG, and TNF-α levels in the blood and liver (Fig. 3 and S1). Treatment with HP1, HP2, or HP3 reduced HFD-induced TC, TG, TNF-α, ALT, AST, and LPS levels in the blood, while the HC level was increased. Furthermore, their treatments suppressed HFD-induced NF-κB activation, iNOS, COX-2, and α-SMA expression and increased claudin-1, and ZO-1 expression in the liver. HFD feeding also induced myeloperoxidase activity, TNF-α, IL-1β, and IL-6 expression, and NF-κB activation in the colon while IL-10, claudin-1, and ZO-1 expressions were suppressed (Fig. 4 and S2). Oral gavage of HP1, HP2, or HP3 suppressed HFD-induced myeloperoxidase activity, NF-κB activation, and TNF-α expression in the colon and induced IL-10 and tight junction protein expression. Treatments also suppressed HFD-induced LPS production and the Proteobacteria population while the Bacteroidetes population was decreased. Overall, of tested bacteria, HP2 alleviated HFD-induced bodyweight gain, liver steatosis, colitis and gut dysbiosis most potently, followed by HP1 and HP3.

Long-term feeding of HFD causes obesity, which increases the well-known risk for the occurrence of insulin resistance, type 2 diabetes, heart disorders, non-alcoholic liver steatosis, gut inflammation, and gut dysbiosis [1, 2, 16]. HFD feeding increases gut Proteobacteria and Firmicutes populations and decreases the gut Bacteriodetes population in humans and mice [6, 7, 17]. In the present study, treatment with IL-10 expression-inducing gut bacteria, particularly HP2, suppressed the HFD-induced Proteobacteria population and LPS production in the gut microbiota. It also suppressed myeloperoxidase activity, NF-κB activation, and TNF-α expression in the colon, and TC, TG, TNF-α, and LPS levels in the blood and liver, while IL-10 and tight junction protein expression levels were increased, resulting in the attenuation of liver steatosis and colitis. Furthermore, treatment suppressed HFD-induced body weight gain. Castro-Rodriguez *et al*. reported that *Leuconostoc mesenteroides* subsp *mesenteroides* SD23 reduced body weight, liver steatosis, and liver IL-10 expression in mice with HFD-induced rats [10]. Jang *et al*. reported that *Lactobacillus sakei* OK67 and PK16 alleviated obesity, anxiety, and colitis through the inhibition of the gut Proteobacteria population, bacterial LPS production, and proinflammatory cytokine expression and induction of IL-10 expression [8]. OK67 and PK16 also inhibited HFD-induced liver steatosis in mice [6]. These findings suggest that IL-10 expression-inducing gut bacteria such as HP1, HP2, and HP3 can suppress HFD-induced liver steatosis and obesity by the attenuation of gut inflammation and dysbiosis.

## Supplementary material

Supplementary data for this paper are available on-line only at http://jmb.or.kr.



## Figures and Tables

**Fig. 1:**
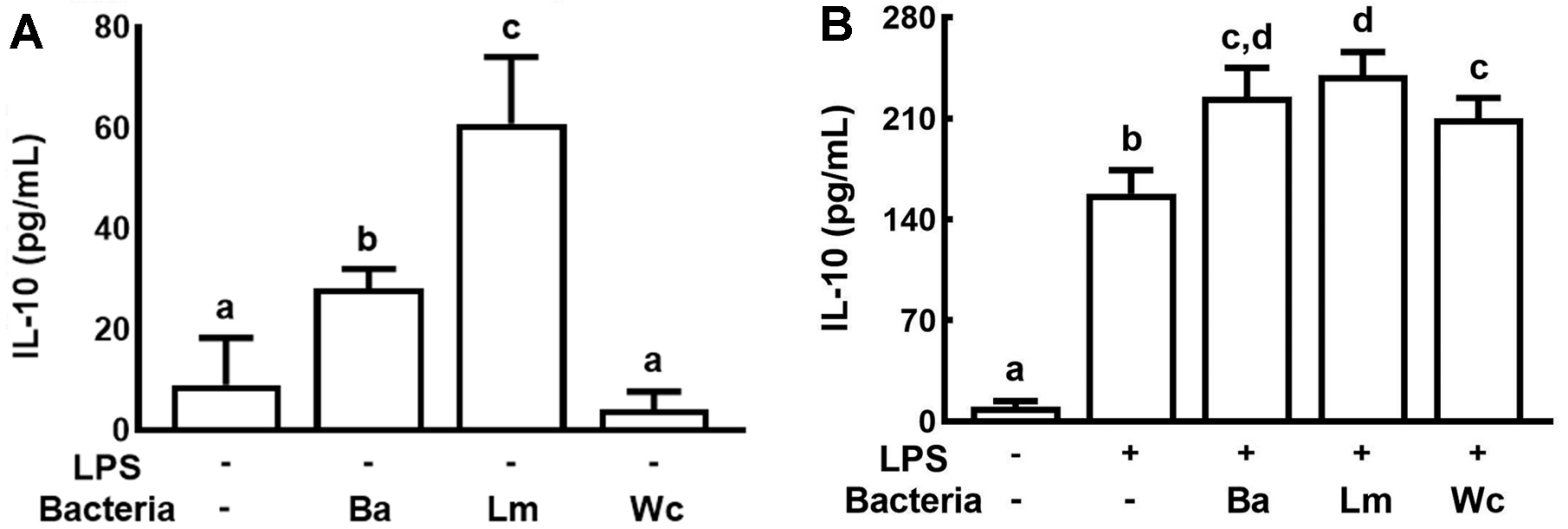
Effects of IL-10 expression-inducing gut bacteria in macrophages. (**A**) Effects in macrophages. (**B**) Effects in LPS-stimulated macrophages. Macrophages were incubated with probiotics (1 × 10^5^ CFU/well) in the absence or presence of LPS for 20 h. IL-10 levels were assessed using ELISA kits. Ba, treated with *Bifidobacterium adolescentis* HP1 (1 × 10^5^ CFU/ml); Lm, treated with *Lactobacillus mucosae* HP2 (1 × 10^5^ CFU/ml); Wc, treated with *Weissella cibaria* HP3 (1 × 10^5^ CFU/ml). Each value is expressed as mean ± SD (*n* = 4). Means with same letters are not significantly different (*p* < 0.05).

**Fig. 2:**
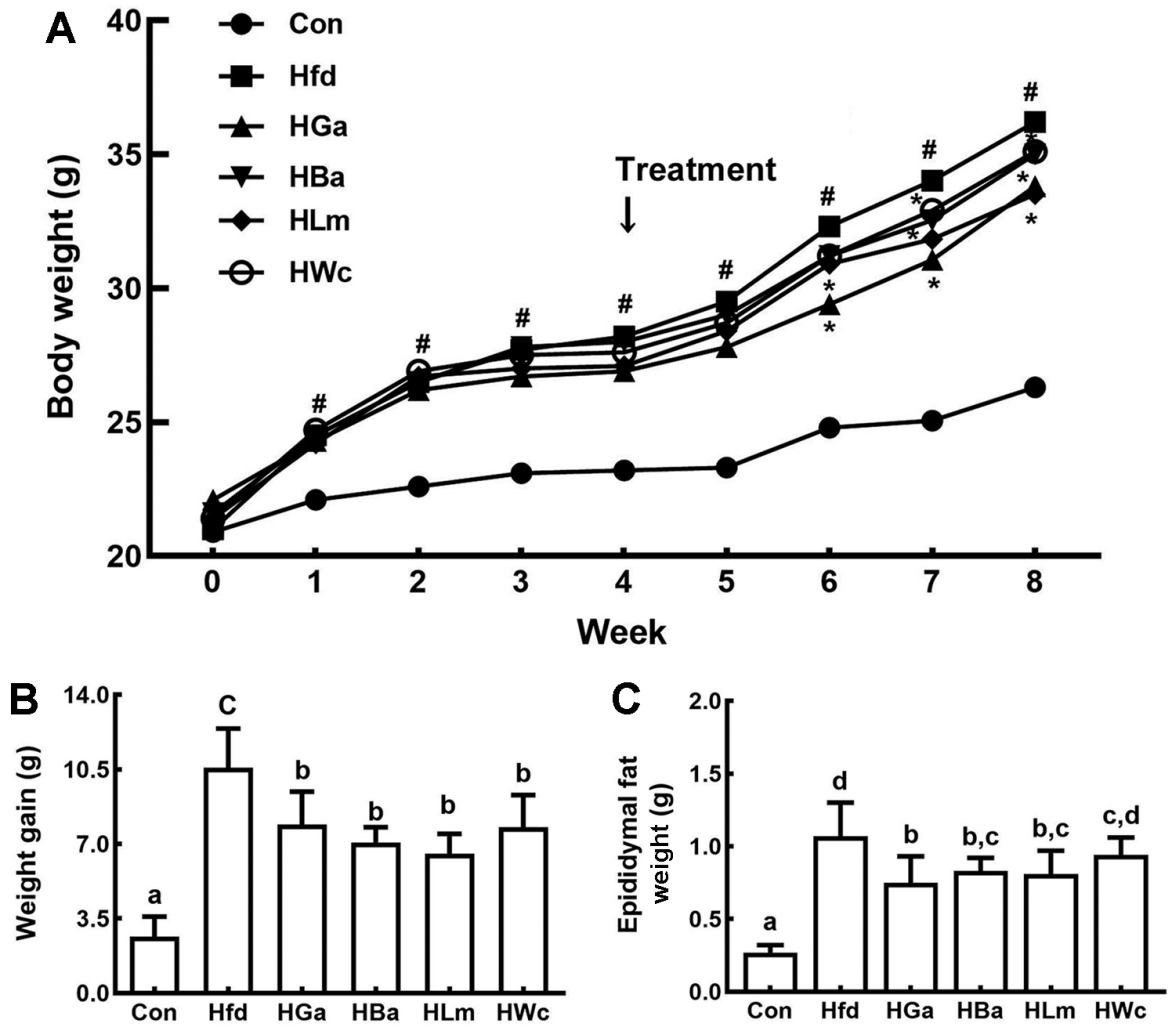
Effects of HP1, HP2, and HP3 on the periodical bodyweight change (A), bodyweight gain (B), and epididymal fat pad weights (C) in mice with HFD-induced obesity. Test agents [Con, vehicle; Hfd, treated with saline in mice with HFD-induced obesity (HIO); HGa, treated with Garcinia (1 mg/kg) in HIO mice; HBa, treated with HP1 (1 × 10^9^ CFU/mouse/day) in HIO mice; HLm, treated with HP2 (1 × 10^9^ CFU/mouse/day) in HIO mice; HWc, treated with HP3 (1 × 10^9^ CFU/mouse/day) in HIO mice] were orally gavaged for 4 weeks. Each value is expressed as mean ± SD (*n* = 10). ^#^*p* < 0.05 vs. Con group. *p < 0.05 vs. Hfd group. Means with same letters are not significantly different (*p* < 0.05).

**Fig. 3:**
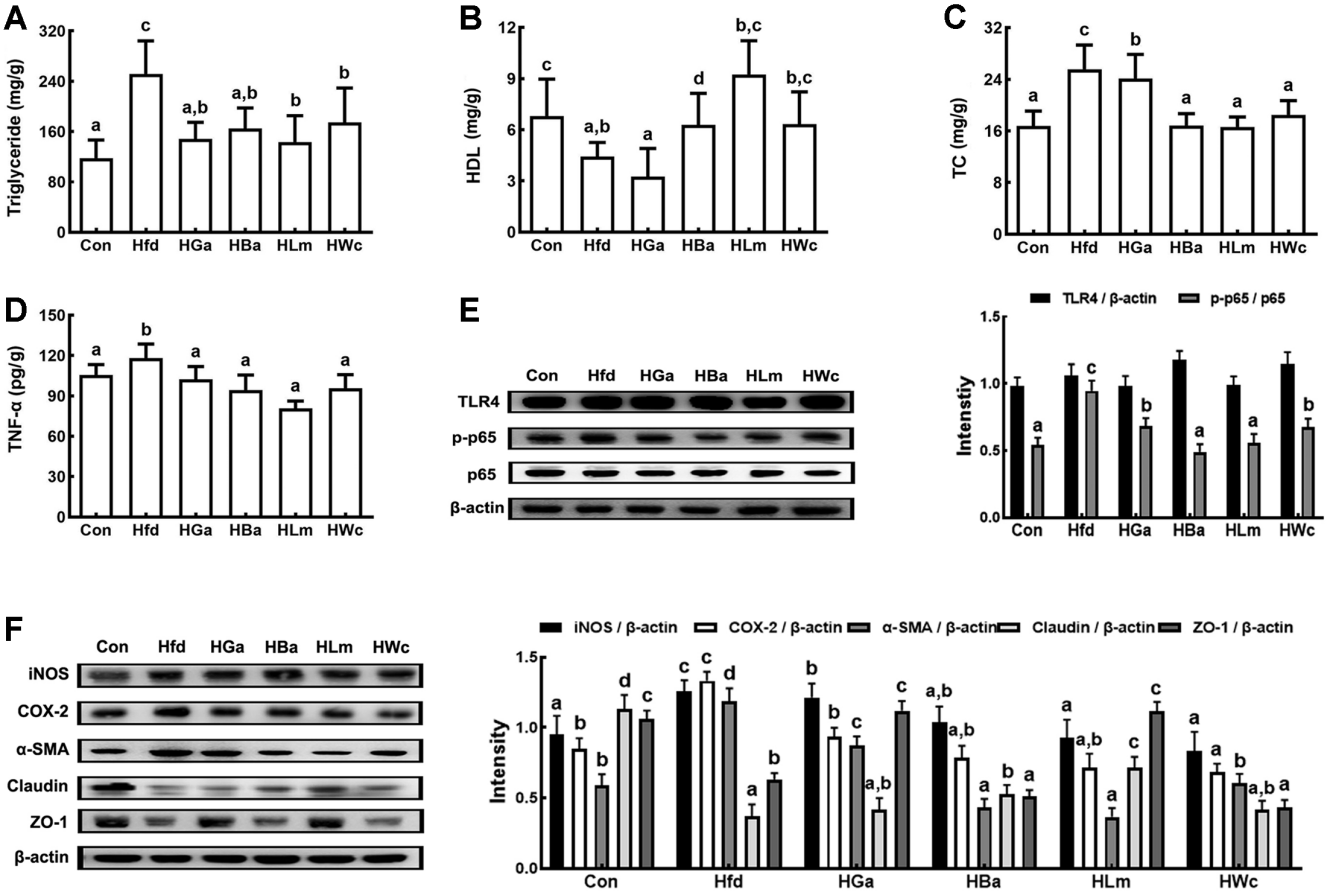
Effects of HP1, HP2, and HP3 on HFD-induced TG (A), HDL (B), TC (C), TNF-α (D), NF-κB activation (E), and iNOS, COX-2, α-SMA, claudin-1, and ZO-1 expression (F) in the liver of mice. Test agents [Con, vehicle; Hfd, treated with saline in mice with HFD-induced obesity (HIO); HGa, treated with Garcinia (1 mg/kg) in HIO mice; HBa, treated with HP1 (1 × 10^9^ CFU/mouse/day) in HIO mice; HLm, treated with HP2 (1 × 10^9^ CFU/mouse/day) in HIO mice; HWc, treated with HP3 (1 × 10^9^ CFU/mouse/day) in HIO mice] were orally gavaged for 4 weeks. Each value is expressed as mean ± SD (*n* = 10). ^#^*p* < 0.05 vs. Con group. *p < 0.05 vs. Hfd group. Means with same letters are not significantly different (*p* < 0.05).

**Fig. 4:**
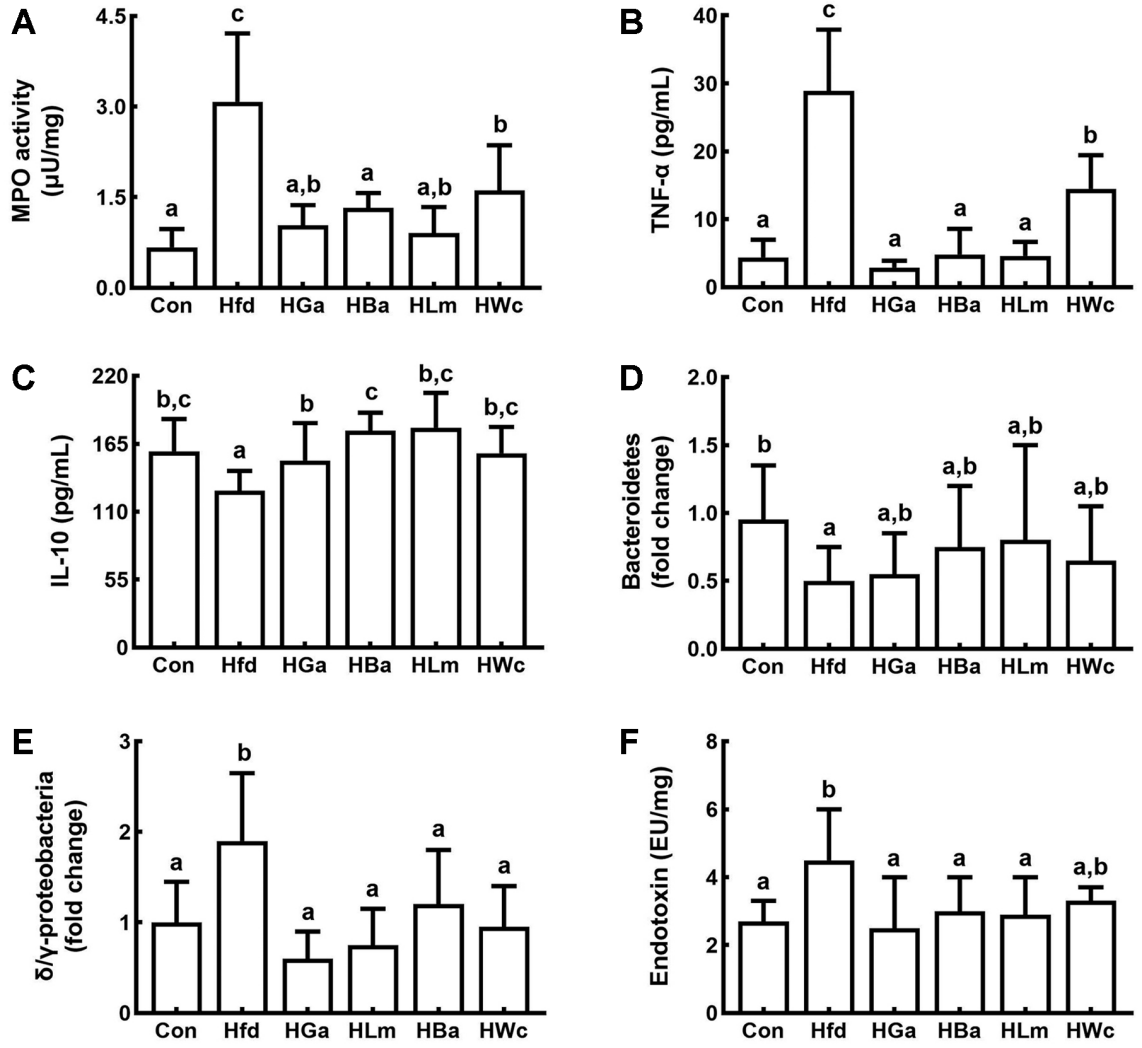
Effects of HP1, HP2, and HP3 on HFD-induced colitis and fecal microbiota alteration in mice. (**B**) Effects on myeloperoxidase (MPO) activity (**A**) and TNF-α (**B**) and IL-10 (**C**) expression. Effects on the Bacteroidetes (**D**) and Proteobacteria populations (**E**) and LPS production (**F**) in the gut microbiota. Test agents [Con, vehicle; Hfd, treated with saline in mice with HFD-induced obesity (HIO); HGa, treated with Garcinia (1 mg/kg) in HIO mice; HBa, treated with HP1 (1 × 10^9^ CFU/mouse/day) in HIO mice; HLm, treated with HP2 (1 × 10^9^ CFU/mouse/day) in HIO mice; HWc, treated with HP3 (1 × 10^9^ CFU/mouse/day) in HIO mice] were orally gavaged for 4 weeks. Each value is expressed as mean ± SD (*n* = 10). ^#^ *p* < 0.05 vs. Con group. *p < 0.05 vs. Hfd group. Means with same letters are not significantly different (*p* < 0.05).
