# Supplementary material for: IL-10 Expression-Inducing Gut Bacteria Alleviate High-Fat Diet-Induced Obesity and Hyperlipidemia in Mice
Source: J Microbiol Biotechnol. 2020 Jan 9;30(4):599–604. doi: 10.4014/jmb.1912.12014 (PMC9728323; doi:10.4014/jmb.1912.12014)
Supplement: Supplementary file 1 [file JMB-30-4-599-supple.pdf]

J. Microbiol. Biotechnol.
https://doi.org/10.4014/jmb.1912.12014

IL-10 expression-inducing gut bacteria alleviate high-fat diet-induced obesity and liver steatosis in mice

Materials and methods

Materials

LPS purified from *Escherichia coli* O111:B4 and RPMI1640 were purchased from Sigma (St. Louis, MO). Enzyme-linked immunosorbent assay (ELISA) kits for TNF-α, IL-1β, IL-6, and IL-10 were purchased from eBioscience (San Diego, CA). Limulus amoebocyte lysate (LAL) assay kit for LPS was purchased from Cape Cod Inc. (E. Falmouth, MA). Antibodies for p65, p-p65, nuclear factor of kappa light polypeptide gene enhancer in B-cells inhibitor-alpha (IκBα), p-IκBα, α-smooth muscle actin (SMA), claudin-1, occludin, zonula occludens (ZO)-1, toll-like receptor (TLR)-4, cyclooxygenase (COX)-2, inducible nitric oxide synthase (iNOS) and β-actin were purchased from Cell Signaling Technology (Beverly, MA). Low-fat (D12450B) and high-fat diets (D12492) (Supplement Table S1) were purchased from Research Diets Inc. (New Brunswick, NJ).

Supplement Table S1. The ingredients of low and high-fat diets used in the present experiment

Ingredient	Low fat diet		High fat diet	
	gm	Kcal	gm	Kcal
Casein, 30 Mesh	189.6	758.3	258.4	1033.8
Corn Starch	298.6	1194.3	0.0	0
Maltodextrin 10	33.2	132.7	161.5	646.1

Sucrose	331.7	1327.0	88.9	355.6
Cellulose, BW200	47.4	0	64.6	0
Soybean Oil	23.7	213.3	32.3	290.8
Lard*	19.0	170.6	316.6	2849.4
L-Cystine	2.8	11.4	3.9	15.5
Mineral mix S10026	9.5	0	12.9	51.7
Dicalcium phosphate	12.3	0	16.8	0
Calcium carbonate	5.2	0	7.1	0
Potassium citrate, 1	15.6	0	21.3	0
Vitamin mix V10001	9.5	37.9	12.9	51.7
Choline bitartrate	1.9	0	2.6	0
FD&C Yellow Dye #5	0.05	0	0.05	0
Total	1000.0	3845.3	1000.0	5294.6

* Supplemented instead of corn starch and sucrose.

Assay of aspartate transaminase (AST), alanine transaminase (ALT), triglyceride (TG), total cholesterol (TC), and high-density lipoprotein cholesterol (HC) in the blood and liver

Livers were homogenized in radio immunoprecipitation assay (RIPA) lysis buffer (Pierce, Rockford, IL, USA) [1]. Liver lysates were centrifuged (10,000 *g,* 10 min, 4°C). Bloods were centrifuged at 1,000 *g* for 10 min. The resulting supernatants were used for the assays of ALT, AST, TG, TC, and HC. Their concentrations were assayed using commercial kits (Asan pharmaceutical Co., Seoul, Korea) [2].

Determination of cytokines and LPS

Livers and colons were homogenized in RIPA lysis buffer at 4°C and centrifuged (10,000 *g,*

10 min, 4°C) [1]. Bloods were centrifuged (1,000 *g,* 10 min). The cytokine levels of their supernatants were assayed using ELISA kits [1].

The contents of LPS in the blood and feces, which were centrifuged (10,000 *g,* 10 min, 4°C) were assayed by using LAL assay kit assay according to the method of Kim et al. [3].

Immunoblotting

The supernatant proteins of tissue lysates (20 μg protein) were separated by 10% SDS-polyacrylamide gel electrophoresis and transferred to polyvinylidenedifluoride membranes [2]. The membranes were serially incubated with antibodies for p-p65, p65, TLR4, IκBα, p-IκBα, iNOS, COX-2, α-SMA, claudin-1, and β-actin (1:1000 diluted) at 4°C overnight and secondary antibody (1:1000 diluted) for 1 h at room temperature. Protein bands were visualized with the enhanced chemiluminescence reagent.

Assays of myeloperoxidase activity

Liver and colon tissues were homogenized in 10 mM potassium phosphate buffer (pH 7.0) containing 0.5% hexadecyl trimethyl ammonium bromide, and centrifuged (13,200 *g*, 10 min, 4°C) according to the method of Jang et al. [2]. The resulting supernatants (50 μL) were added in the reaction mixture containing 0.1 mM H_2O_2 and 1.6 mM tetramethyl benzidine, incubated at 25°C, and monitored the absorbance at 650 nm for 5 min.

Quantitative real time-polymerase chain reaction (qPCR)

Total DNA was purified from mouse feces according to the method of Lee et al. [4]. qPCR for gut bacteria was carried out with SYBR premix in a Takara thermal cycler according to

the method of Lim et al. [5]. The thermal cycling was performed at 95°C for 30 s by 42 cycles of denaturation at 95°C for 5 s and amplification at 63°C for 30 s. Gut bacteria levels were calculated, relative to bacterial 16S rDNA level. Primers are shown in Supplement Table S2.

Supplement Table S2. Primers for qPCR

Gene	Primer	
	forward	Reverse
16S rDNA	5'-TCG TCG GCA GCG TCA GAT GTG TAT AAG AGA CAG GTG CCA GCM GCC GCG GTA A-3'	5'-GTC TCG TGG GCT CGG AGA TGT GTA TAA GAG ACA GGG ACT ACH VGG GTW TCT AAT-3'
Firmicutes	5'-GGA GYA TGT GGT TTA ATT CGA AGC A-3'	5'-AGC TGA CGA CAA CCA TGC AC-3'
Bacteroidetes	5'-AAC GCG AAA AAC CTT ACC TAC C-3'	5'-TGC CCT TTC GTA GCA ACT AGT G-3'
δ/γ-Proteobacteria	5'-GCT AAC GCA TTA AGT RYC CCG-3'	5'-GCC ATG CRG CAC CTG TCT-3'
Actinobacteria	5'-TGT AGC GGT GGA ATG CGC-3'	5'-AAT TAA GCC ACA TGC TCC GCT-3'

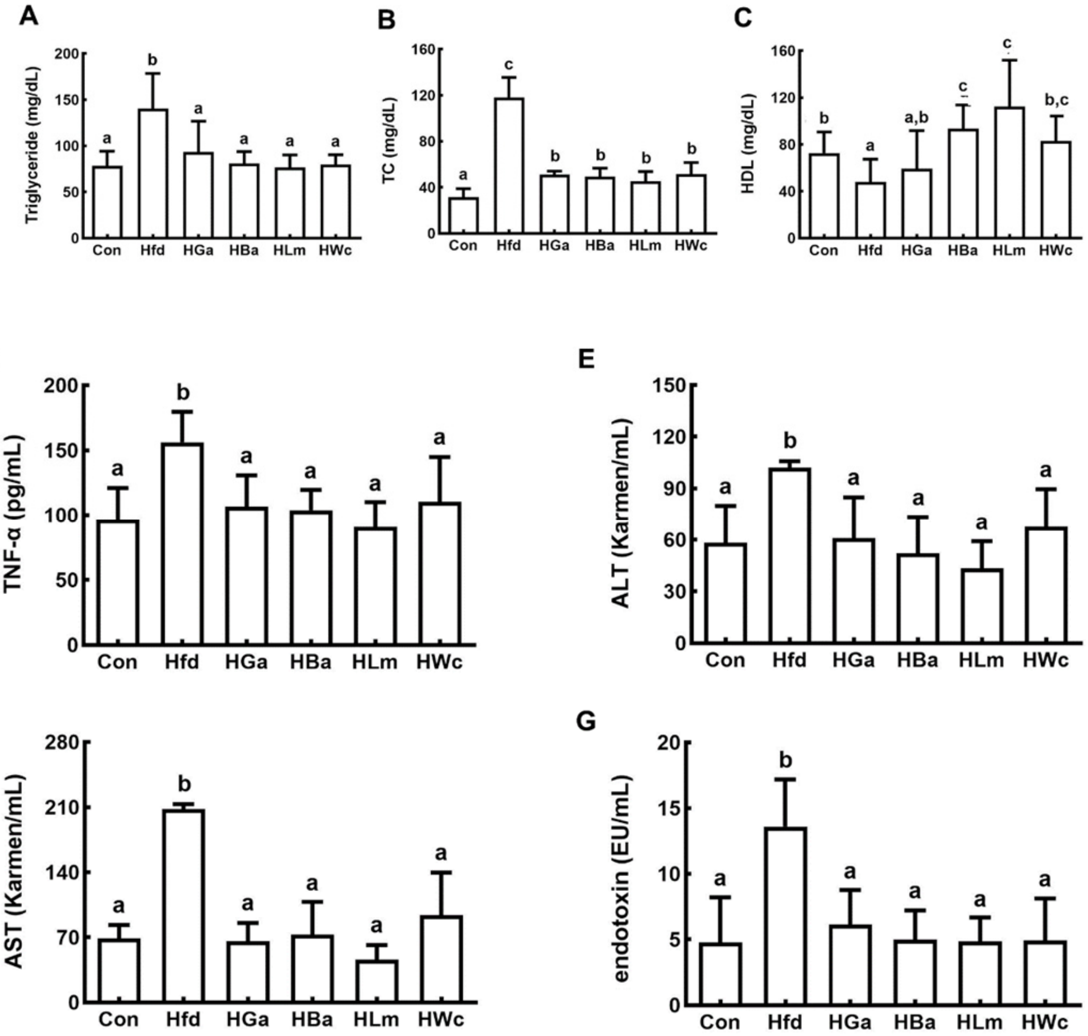

Supplement Figure S1. Effects of HP1, HP2, and HP3 on the blood triglyceride (A), total cholesterol (TC, B), high-density lipoprotein cholesterol (HDL, C), TNF-α (D), alanine transaminase (ALT, E), aspartate transaminase (AST, F), and endotoxin levels (G) in mice with HFD-induced obesity. Test agents [Con, vehicle; Hfd, treated with saline in mice with HFD-induced obesity (HIO); HGa, treated with Garcinia (1 mg/kg) in HIO mice; HBa, treated with HP1 (1×10^9 CFU/mouse/day) in HIO mice; HLm, treated with HP2 (1×10^9 CFU/mouse/day) in HIO mice; HWc, treated with HP3 (1×10^9 CFU/mouse/day) in HIO

mice] were orally gavaged for 4 weeks. Each value is expressed as mean ± SD (n=10). $^{\#}$ p<0.05 vs. Con group. *p < 0.05 vs. Hfd group.

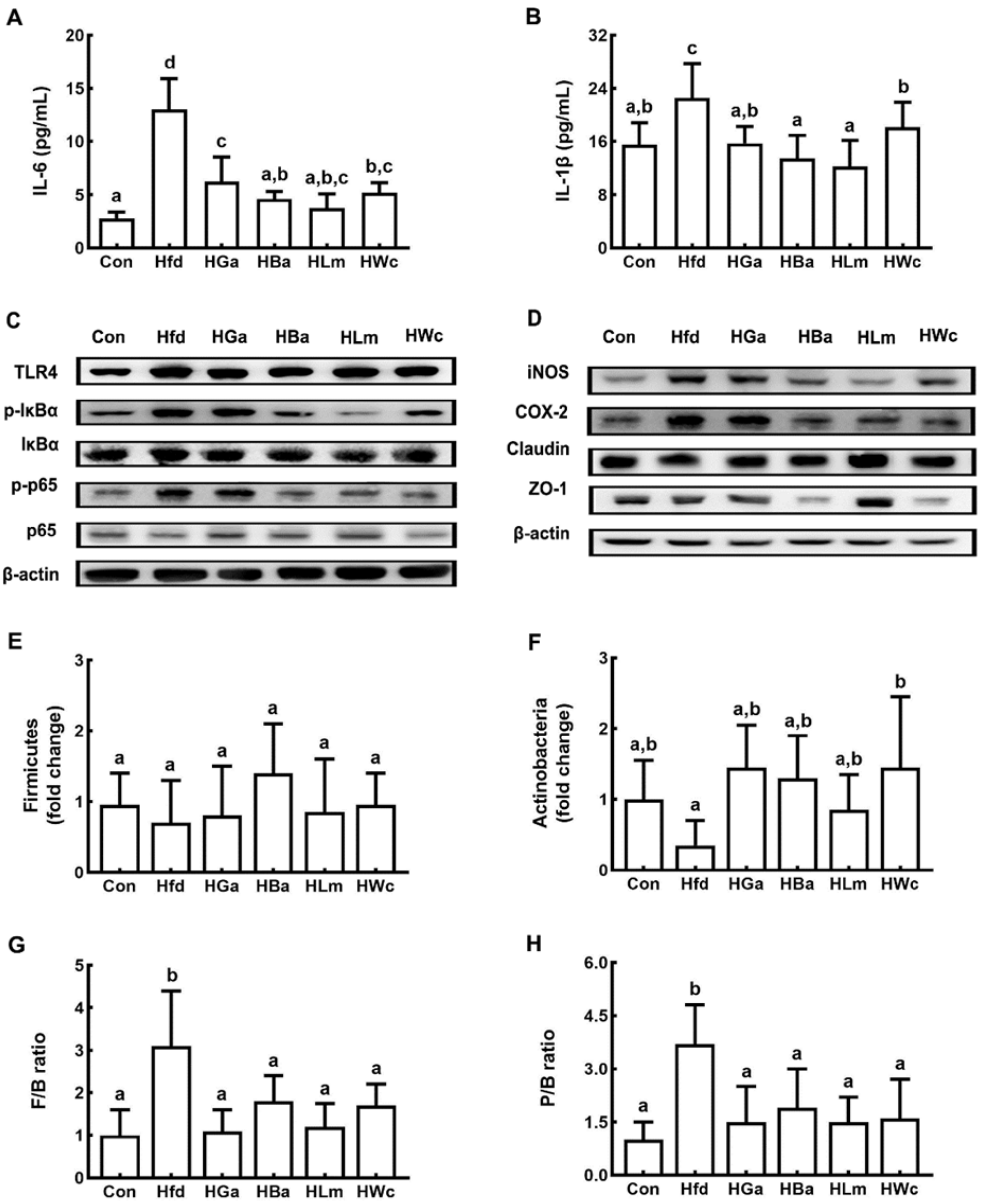

Supplement Figure S2. Effects of HP1, HP2, and HP3 on HFD-induced colitis and fecal microbiota alteration in mice. Effects on IL-6 (A) and IL-1β (B) expression in the colon. (C) Effects on the activation of NF-κB signaling molecules in the colon. (D) Effects on iNOS,

COX-2, claudin-1, and ZO-1 expression in the colon. (E) Effects on the fecal Firmicutes population. (F) Effects on the fecal Actinobacteria population. (G) Effects on the ratio of Firmicutes to Bacteroidetes population. (H) Effects on the ratio of Proteobacteria to Bacteroidetes population. Test agents [Con, vehicle; Hfd, treated with saline in mice with HFD-induced obesity (HIO); HGa, treated with garcinia (1 mg/kg) in HIO mice; HBa, treated with HP1 (1×10^9 CFU/mouse/day) in HIO mice; HLm, treated with HP2 (1×10^9 CFU/mouse/day) in HIO mice; HWc, treated with HP3 (1×10^9 CFU/mouse/day) in HIO mice] were orally gavaged for 4 weeks. Gut microbiota composition was analyzed by qPCR. Each value is expressed as mean ± SD (n=10). # $p<0.05$ vs. Con group. *$p < 0.05$ vs. Hfd group.

References

[1] Jang HM, Han SK, Kim JK, Oh SJ, Jang HB, Kim DH. 2019. Lactobacillus sakei alleviates high-fat-diet-induced obesity and anxiety in mice by inducing AMPK activation and SIRT1 expression and inhibiting gut microbiota-mediated NF-κB activation. *Mol. Nutr. Food Res.* 63:e1800978.

[2] Jang SE, Jeong JJ, Kim JK, Han MJ, Kim DH. 2018. Simultaneous amelioration of colitis and liver injury in mice by Bifidobacterium longum LC67 and Lactobacillus plantarum LC27. *Sci. Rep.* 8:7500.

[3] Kim KA, Gu W, Lee IA, Joh EH, Kim DH. 20122. High fat diet-induced gut microbiota exacerbates inflammation and obesity in mice via the TLR4 signaling pathway. *PLoS One* 7: e47713.

[4] Lee HJ, Jeong JJ, Han MJ, Kim DH. 2018. Lactobacillus plantarum C29 alleviates TNBS-

induced memory impairment in mice. *J. Microbiol. Biotechnol.* 28(1):175-179.

[5] Lim SM, Choi HS, Kim DH. 2017. The mixture of Anemarrhena asphodeloides and Coptidis chinensis attenuates high-fat diet-induced colitis in mice. *Am. J. Chin. Med.* 45:1033-1046.
